# Draft genomes of five putative biosurfactant-producing *Serratia* sp. isolates from Laguna de Bay, Philippines

**DOI:** 10.1128/mra.01268-24

**Published:** 2025-03-10

**Authors:** Nacita B. Lantican, Glaezel Angelique T. Barredo, Albert R. Rosana, Ann Clarisse S.V. Siababa, Andrew D. Montecillo

**Affiliations:** 1 Microbiology Division, Institute of Biological Sciences, College of Arts and Sciences, University of the Philippines Los Banos, Los Banos, Laguna, Philippines; 2 Genetics and Molecular Biology Division, Institute of Biological Sciences, College of Arts and Sciences, University of the Philippines Los Banos, Los Banos, Laguna, Philippines; University of Southern California, Los Angeles, California, USA

**Keywords:** Laguna de Bay, *Serratia*, biosurfactant, whole-genome sequencing, WGS, draft genomes

## Abstract

Here, we report on the draft genomes of five putative biosurfactant-producing isolates of *Serratia* sp. from the East, West, and South bays of Laguna de Bay, Philippines. The contigs ranged from 20 to 21, and the genome lengths ranged from 5,175,553 to 5,214,031 bp.

## ANNOUNCEMENT

Laguna de Bay is the largest inland wetland lake in the Philippines located around Metro Manila and extends to the province of Rizal and Laguna. It is extensively being used for fishing, source of food, and for irrigation. The extent of pollution is, however, very high with diverse sources of pollutants from household, agriculture, and industry; hence, it is expected to have high concentrations of hydrocarbons (1). The presence of biosurfactant-producing microorganisms would be an important factor in bioremediating such polluted bodies of water.

Five putative biosurfactant-producing bacterial strains were isolated from sediments and water samples from various sites (East, West, and South bays) in Laguna de Bay (14.3935° N, 121.1939° E) (2) following the method of Shoeb et al. (3). Briefly, for each sample, 1 g of sediment or 1 mL of lake water samples was diluted 10-fold using maximum recovery diluent (0.85% NaCl and 0.15% peptone). Dilutions were plated on nutrient agar plates and incubated at 30°C for 24 to 48 h. Single-colony isolates were subcultured on the same medium until axenic cultures were obtained and confirmed microscopically. Cell pellets were sent to Macrogen, Inc., South Korea for 16S rRNA gene and whole-genome sequencing. Sample preparation and DNA extraction were performed following the standard procedures of Macrogen Identification Services. The boiling method using Instagene matrix was used to extract the total DNA, followed by PCR amplification and sequencing. For whole-genome sequencing, TruSeq DNA Nano Library Preparation Kit (Illumina, San Diego, CA) and 2 × 150 bp cycle were used in Illumina NovaSeq 6000 (Illumina, San Diego, CA).

**Fig 1:**
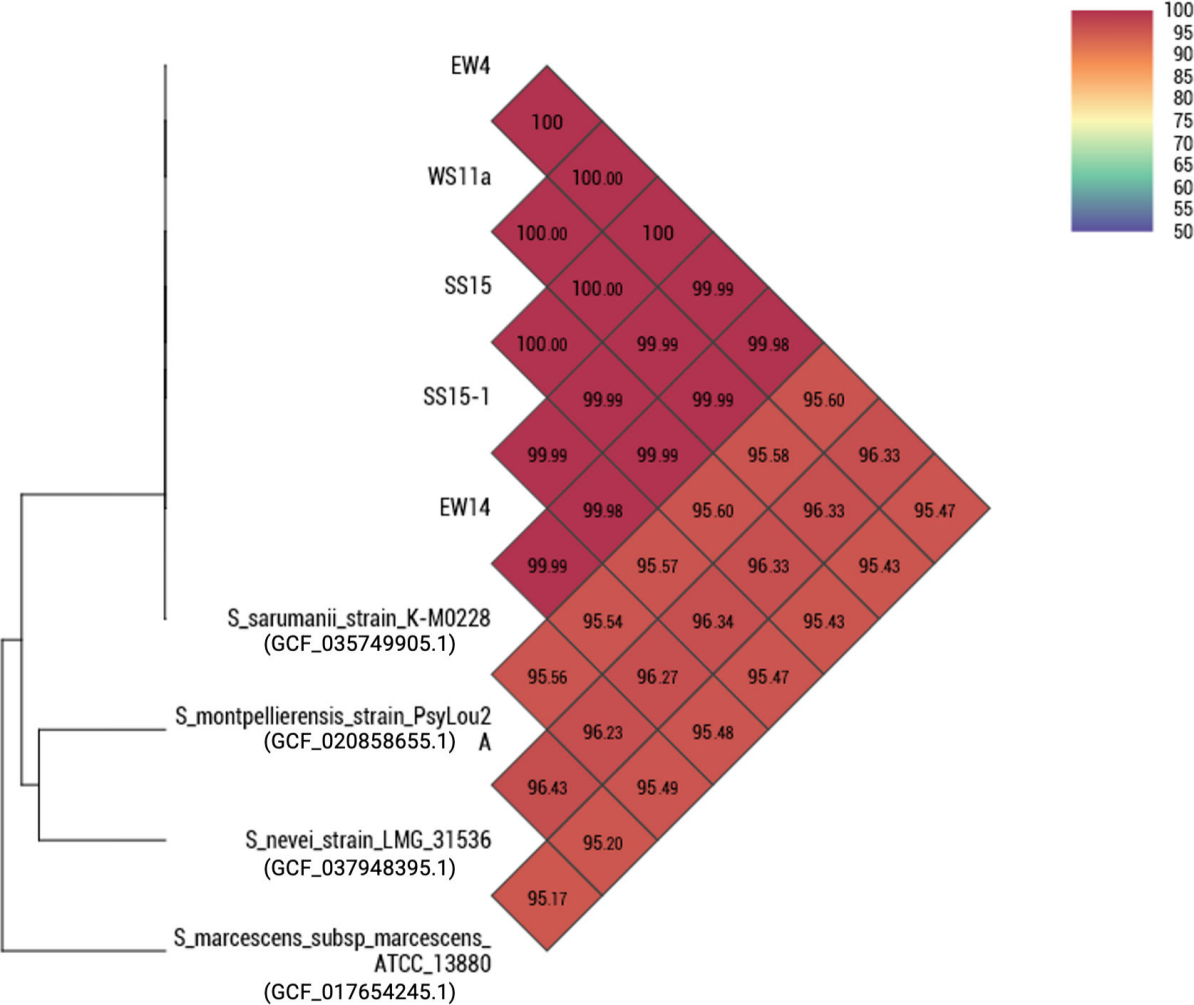
Pairwise average nucleotide identity (ANI) analysis of pucbitative biosurfactant-producing *Serratia* sp. isolates from Laguna de Bay and select reference *Serratia* genomes.

Paired-end raw reads were assessed and trimmed using fastp v.0.23.4 (4) with default parameters. *De novo* assembly was done in Unicycler v.0.5.1 (5) with default parameters, and assembly quality was assessed using QUAST v4.6 (6). Contigs with less than 500 bp length were discarded. The draft genome sequences were annotated using the National Center for Biotechnology Information (NCBI) Prokaryotic Genome Annotation Pipeline (PGAP) (7), and taxonomic placement of the isolates was established using the Microbial Genomes Atlas tool (8) by 16S SSU rRNA classification based on RDP 16S rRNA training set No. 19 07/2023 and RDP Naive Bayesian rRNA Classifier Version 2.14 and by calculating the average nucleotide identity (ANI) in comparison to entries in the NCBI TypeMat database (release 2024-08). Assembly completeness was determined in BUSCO v.5.8.0 (9) with bacteria_odb10 lineage data set. Default parameters were used with all tools, unless otherwise specified. GenBank and SRA accession numbers, sequencing, assembly, and annotation information for each isolate were summarized in Table 1. Digital DNA–DNA hybridization (dDDH) values (formula 2) were calculated against the nearest genome match using the recommended settings in GGDC 4.0 (10). Pairwise ANI and GGDC were calculated in OrthoANI v.0.93.10 (11). The nearest matching species for all genomes is *Serratia sarumanii* (99.9% ANI, 100% dDDH) (Fig. 1).

**TABLE 1:** GenBank and Sequencing Read Archives accession numbers, sequencing library and genome assembly statistics, and annotation of five (5) putative biosurfactant-producing isolates from Laguna de Bay, Philippines

Isolate code	Location and sample type	Whole-genome sequence accession	SRA accession number	Genome size (bp)	Gc (%)	Filtered reads used in the assembly	Mean depth (x)	Contigs	N50 (bp)	Predicted total genes, total CDS, and total protein- coding genes^*a*^	BUSCO completeness (%)	Nearest genome match
EW14	East Bay—water	JBJHGL000000000	SRR31327912	5,175,553	60	48,466,944	1,414	21	2,618,309	4,920; 4,832; 4,806	95.2	*Serratia sarumanii* K-M0706 (GCF_029962605.1)
EW4	East Bay—water	JBJHGK000000000	SRR31327911	5,214,031	60	41,897,142	1,213	20	2,886,712	4,982; 4,892; 4,866	95.2	*Serratia sarumanii* K-M0706 (GCF_029962605.1)
SS15	South Bay—sediment	JBJHGJ000000000	SRR31327910	5,213,867	60	48,884,526	1,416	20	2,886,724	4,980; 4,892; 4,866	95.2	*Serratia sarumanii* K-M0706 (GCF_029962605.1)
SS15-1	South Bay—sediment	JBJHGI000000000	SRR31327909	5,213,181	60	45,959,424	1,331	21	2,886,712	4,978; 4,890; 4,864	95.2	*Serratia sarumanii* K-M0706 (GCF_029962605.1)
WS11a	West Bay—sediment	JBJHGH000000000	SRR31327908	5,213,854	60	47,766,626	1,383	20	2,886,712	4,980; 4,892, 4,866	95.2	*Serratia sarumanii* K-M0706 (GCF_029962605.1)

^*a*^ Genomes were annotated using the NCBI PGAP (7).

## Data Availability

The sample and sequencing information were uploaded to the NCBI SRA under BioProject accession number PRJNA1185677. The accession numbers for all five SRA files are as follows: SRR31327912, SRR31327911, SRR31327910, SRR31327909, and SRR31327908. This Whole Genome Shotgun project has been deposited in GenBank under accession nos. JBJHGL000000000, JBJHGK000000000, JBJHGJ000000000, JBJHGI000000000, and JBJHGH000000000. The version described in this paper is the first version.
